# Effect of Dissolved Oxygen Concentration on the Microbiologically Influenced Corrosion of Q235 Carbon Steel by Halophilic Archaeon *Natronorubrum tibetense*

**DOI:** 10.3389/fmicb.2019.00844

**Published:** 2019-04-25

**Authors:** Hongchang Qian, Pengfei Ju, Dawei Zhang, Lingwei Ma, Yuting Hu, Ziyu Li, Luyao Huang, Yuntian Lou, Cuiwei Du

**Affiliations:** ^1^ Beijing Advanced Innovation Center for Materials Genome Engineering, Institute for Advanced Materials and Technology, University of Science and Technology Beijing, Beijing, China; ^2^ Shanghai Aerospace Equipment Manufacturer, Shanghai, China

**Keywords:** carbon steel, archaea, biofilm, microbiological influenced corrosion, oxygen concentration

## Abstract

The influence of dissolved oxygen concentration (DOC) on the microbiologically influenced corrosion (MIC) of Q235 carbon steel in the culture medium of halophilic archaeon *Natronorubrum tibetense* was investigated. The increase of DOC from 0.0 to 3.0 ppm was found to strengthen the oxygen concentration cell by promoting cathodic reaction. Meanwhile, the increased DOC also promoted archaeal cell growth, which could consume more metallic iron as energy source and aggravated the localized corrosion. When the DOC further increased to 5.0 ppm, the uniform corrosion was dominant as the biofilms became uniformly presented on the steel surface. Combined with the stronger inhibition effect of oxygen diffusion by the increased biofilm coverage, the MIC of carbon steel in the 5.0 ppm medium was weaker than that in the 3.0 ppm medium. From weight loss and electrochemical tests, the results all demonstrated that the carbon steel in the 3.0 ppm medium had the largest corrosion rate.

## Introduction

Microbiologically influenced corrosion (MIC) is mainly caused by the formation of biofilm on metal surface. Metabolic activity of microorganisms in biofilm and physical barrier of extracellular polymeric substance (EPS) will affect the electrochemical process of metals and then lead to their corrosion acceleration or inhibition (Videla and Herrera, 2005; Little et al., 2008; Stadler et al., 2008). MIC has become one of the main threats in the field of oil and gas industry, water treatment industry, and marine engineering, which brings huge economic losses and potential safety hazards (Li et al., 2017). For a long time, numerous research works have been focused on sulfate reducing bacteria (SRB), iron oxidizing bacteria (IOB), sulfur oxidizing bacteria (SOB), and other bacteria (Enning and Garrelfs, 2014; Huber et al., 2016; Liang et al., 2016; Liu et al., 2016; Xu et al., 2016, 2018; Jia et al., 2017; Karn et al., 2017; Dou et al., 2018; Liu and Cheng, 2018). For the ubiquitous microorganisms in the natural environment, such as SRB, their MIC mechanisms have been studied in-depth, and comprehensive corrosion mechanisms have been well established (Venzlaff et al., 2013; Rajala et al., 2015; Li et al., 2018; Jia et al., 2019). Besides, some researches on MIC have investigated eukaryotic microorganisms in humid atmospheric environment (Qu et al., 2015; Pramila and Ramesh, 2017).

At present, the microbial community is mainly divided into three categories (Woese et al., 1990). In addition to bacteria and eukaryote, archaeon is another important microbial community. Because of its special cell structure and metabolic mode, most archaeal microorganisms must inhabit extreme natural environment, such as methanogenic archaea in strictly anaerobic oil extraction fluid (Duncan et al., 2009), thermoacidophilic archaea in hot spring, and halophilic archaea in saline soil (Liu et al., 2015a; Song et al., 2015). Some archaeal microorganisms have been utilized in industrial production and energy exploitation (Litchfield, 2011; Zhu et al., 2013). Hence, the MIC of metals caused by archaeal microorganisms has begun to attract attention. In the 1990s, the hyperthermophilic archaeon *Archaeoglobus fulgidus* was isolated from oil fields in the North Sea and was shown to be able to cause corrosion of metal equipment for oil and gas exploration (Stetter et al., 1993). In the following decades, some other methanogenic and thermophilic archaea were separated from the oil field system (Davidova et al., 2012; Mand et al., 2016). For example, the thermophilic archaeon, *Thermococcales* sp., which was isolated from Alaskan North Slope (ANS) oil reservoirs, was found to be able to promote corrosion of carbon steel by reducing Fe^3+^ to Fe^2+^ and producing corrosive organic fatty acids (Davidova et al., 2012). In addition, marine rust also contained rich methanogenic archaea. Usher et al. reported that methanogenic archaeon *Methanococcus maripaludis* could promote the corrosion of carbon steel by uptaking electrons directly from carbon steel (Usher et al., 2014). Nevertheless, except for methanogenic and thermophilic archaea, the MIC of other types of archaea has not been noticed so far.

Halophilic archaeon is another kind of important archaeal microorganism. The living environment with high salt concentration is required by halophilic archaea to maintain cell integrity. Halophilic archaeon is widely distributed on the earth. For example, there are vast saline soils in western China, and dozens of halophilic archaea have been found to inhabit these soils (Xu et al., 1999; Liu et al., 2013). Because of the extreme high salinity, halophilic archaea become the main microorganism in these alkali soils. Furthermore, thousands of kilometers of steel pipelines or railways have traversed such soil environments. Whether this type of archaea can affect the corrosion of metal materials and how it functions need to be studied. In our previous research, the effect of halophilic archaea on the corrosion behavior of carbon steel was studied for the first time (Qian et al., 2018). The halophilic archaeon *Natronorubrum tibetense* can promote the corrosion of carbon steel by using carbon steel as energy source. According to the experimental results, the dissolved oxygen in the culture medium had a significant influence on the MIC of carbon steel. Dissolved oxygen not only supplied cell respiration and proliferation but also acted as cathode depolarizer of the corrosion electrochemical process on the steel surface. Therefore, the change of dissolved oxygen concentration (DOC) may affect the MIC behavior of carbon steel.

The purpose of this article is to investigate the effect of DOC on MIC of carbon steel caused by halophilic archaeon *N. tibetense*. The effect of DOC on the corrosion morphology of carbon steel was observed by scanning electron microscopy (SEM) and confocal laser scanning microscopy (CLSM). The effect of DOC on the corrosion electrochemical process of carbon steel was characterized by electrochemical impedance spectroscopy (EIS) and potentiodynamic polarization measurements. The influence of DOC on the composition of corrosion products was analyzed by X-ray photoelectron spectroscopy (XPS). Finally, we discussed the mechanism for the effect of DOC on the MIC of carbon steel by archaeon *N. tibetense*.

## Materials and Methods

### Material, Microorganism, and Culture Medium

The Q235 carbon steel samples (10 mm × 10 mm × 2 mm) were used to conduct the immersion tests. Before immersion tests, the samples were sealed with epoxy resin, leaving only one exposed surface (10 mm × 10 mm). The exposed surfaces were polished by abrasive papers until 1,000 grit, followed by cleaning with deionized water and ethanol. Then, the samples were exposed to UV light for 20 min for sterilization.

The pure archaeon *N. tibetense* strain and its culture medium were provided by China General Microbiological Culture Collection Center (CGMCC). The culture medium contains the following components in 100 ml deionized water: 0.25 g glutamic acid, 0.3 g sodium citrate, 1.5 g casamino acid, 0.2 g KCl, 0.25 g MgSO_4_·7H_2_O, and 25.0 g NaCl. After adjusting the pH value to 8.5 by sterile Na_2_CO_3_ solution, the culture medium undergoes autoclaved treatment for 30 min at 115°C. Then, the *N. tibetense* strain was inoculated into the culture medium and cultivated at 37°C in a shaker. The optical density at 600 nm (OD_600_) values of the culture media were measured by ultraviolet spectrophotometer (Thermo Fisher, Bio Mate3S). The pH values of the media were recorded by pH meter (METTLER TOLEDO, S220-B).

In the immersion tests, the sterilized carbon steel samples were immersed into the sterile and *N. tibetense*-inoculated culture media for 3, 7, and 14 days. In normal culture medium, the DOC was 1.5 ppm, which was measured by a dissolved oxygen meter (Hengxin Instrumentation Co., Ltd., AZ8403). The DOC was adjusted to 0.0, 0.5, 3.0, and 5.0 ppm by injecting pure nitrogen or pure oxygen into the culture media, and the experimental solutions were denoted as 0.0 ppm inoculated medium, 0.5 ppm inoculated medium, 3.0 ppm inoculated medium, and 5.0 ppm inoculated medium, respectively.

### Surface Analysis

The corrosion morphologies before and after removing the corrosion products were characterized by scanning electron microscopy (SEM, FEI Quanta 250). Before SEM observation, the carbon steel samples were immersed into 2.5% (v/v) glutaraldehyde solution for 8 h to immobilize the biofilms. Subsequently, the samples were dehydrated by 50, 60, 70, 80, 90, and 100% ethanol solutions (Qian et al., 2017a, 2019). The sample surfaces were sputter coated with Au to ensure good surface conductivity. The contour and size of the corrosion pits were observed and measured by confocal laser scanning microscopy (CLSM, KEYENCE VK-X). The chemical composition of the corrosion products was analyzed by X-ray photoelectron spectroscopy (XPS, Thermo escalab 250Xi).

### Weight Loss Analysis

After cleaning, drying, and sterilizing, the original weights of the samples were weighed using the electronic analytical balance (METTLER TOLEDO, ME204T/02) with the precision of ±0.1 mg. After 3, 7, and 14 days of immersion tests, the carbon steel samples were taken out from the culture media and rinsed by deionized water. Then, the corrosion products and biofilms were removed from the carbon steel surfaces after 3 min of ultrasonic cleaning in the de-rusting solution (ISO 8407: 2009, IDT). After that, the samples were cleaned with deionized water and ethanol. Subsequently, the weights of the dried samples were weighed again by electronic analytical balance. Triplicate samples were adopted for each test.

### Electrochemical Tests

The electrochemical station (Gamry, Reference 600 Plus) was used to conduct all electrochemical tests. In the electrochemical measurement system, a typical three electrode system was adopted: the sealed carbon steel sample was used as working electrode; the platinum electrode and the saturated calomel electrode (SCE) were used as counter electrode and reference electrode, respectively. The electrochemical impedance spectroscopy (EIS) was measured from 100 kHz to 10 mHz with a sinusoidal perturbation of 10 mV. The potentiodynamic polarization measurement was carried out with the scanning rate of 0.166 mV/s, and the scanning potential ranging was set from −200 to 200 mV vs. open circuit potential (OCP). By extrapolating the linear anode and cathodic Tafel regions, the corrosion current density (*I*_corr_) can be obtained. All electrochemical measurements were performed in triplicate.

## Results

### Growth Process Under Different DOCs

**Figure 1:**
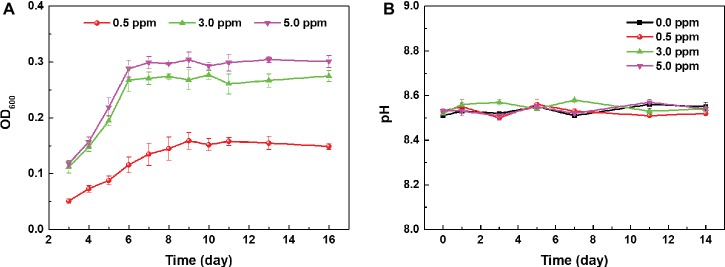
**(A)** Growth curves of archaeon *N. tibetense* in inoculated media with different DOCs and **(B)** the evolution of pH values of different inoculated media with time.

Figure 1A shows the growth curves of archaeon *N. tibetense* in inoculated media with different DOC. From 0 to 6 days, archaeon *N. tibetense* grew and proliferated rapidly, exhibiting exponential growth stage. After 6 days, the growth process of archaeon *N. tibetense* reached a plateau. For aerobic microorganisms, organic substances in the culture medium are consumed by microbial cells as electron donors, and dissolved oxygen is used as electron acceptor to sustain cell respiration. The cell concentration of the stationary phase is determined by the supply amounts of organic substances and DOC. Hence, with the increase of DOC, the concentrations of *N. tibetense* cells during the stationary phase increased gradually. In anaerobic media, archaeon *N. tibetense* was inactive (Qian et al., 2018). The evolution of pH values of different inoculated media during 14 days was illustrated in Figure 1B. During the whole immersion process, the pH values of different media were almost constant and maintained at around 8.5, which were similar to the initial pH value of the *N. tibetense* culture medium. Despite the gradual increase of DOC, no acid metabolite was produced in the culture medium.

### Corrosion Morphologies

**Figure 2:**
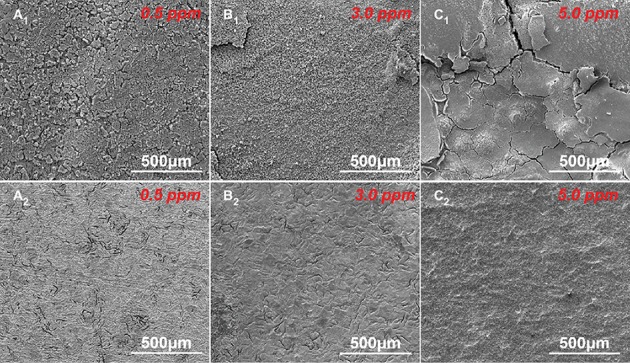
SEM images of sample surfaces **(A_1_–C_1_)** before and **(A_2_–C_2_)** after removing the corrosion products after 14 days of immersion in sterile media with different DOCs.

The corrosion morphologies before and after removing corrosion products after 14-day immersion in sterile media with different DOCs are shown in Figure 2. In our previous study, we have studied the corrosion behavior of carbon steel in the culture medium under anaerobic condition, and found that almost no corrosion occurred after 14 days (Qian et al., 2018). While in sterile media with dissolved oxygen, obvious corrosion appeared on the carbon steel surfaces due to the introduction of oxygen (Figure 2). After 14 days of immersion, the sample surfaces in different media were uniformly covered by corrosion product layers. After removing the corrosion products, all sample surfaces exhibited typical uniform corrosion characteristics.

**Figure 3:**
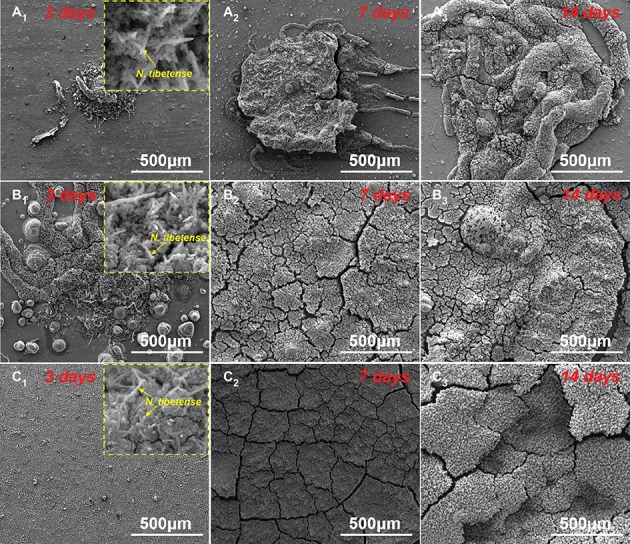
SEM images of the corrosion products on sample surfaces after 3 **(A_1_–C_1_)**, 7 **(A_2_–C_2_)**, and 14 days **(A_3_–C_3_)** of immersion in **(A)** 0.5 ppm, **(B)** 3.0 ppm, and **(C)** 5.0 ppm inoculated media. The insets are the morphologies of *N. tibetense* cells in corrosion products after 3 days.

Figure 3 shows the SEM morphologies of corrosion products on sample surfaces after 3, 7, and 14 days of immersion in inoculated media with different DOCs. For the inoculated medium without DOC, aerobic *N. tibetense* could not grow, so the sample surface was free from obvious corrosion (Qian et al., 2018). When the DOC was 0.5 ppm, a small amount of corrosion product clusters appeared on the sample surface after 3-day immersion (Figure 3A_1_). With the increase of immersion time, the scale of corrosion product clusters expanded gradually (Figure 3A_3_). When the DOC increased to 3.0 ppm, dense corrosion product clusters have formed on the sample surface after only 3 days (Figure 3B_1_). After 7-day immersion, the sample surface was completely covered by corrosion products. For the 5.0 ppm inoculated medium, in the initial stage of immersion, the flat corrosion products have covered the whole sample surface (Figure 3C_1_), and they became thicker along with the immersion time. In addition, some *N. tibetense* cells can be observed in the corrosion products of different media after 3 days, which suggest that the biofilms had begun to affect the corrosion of carbon steels on the third day.

**Figure 4:**
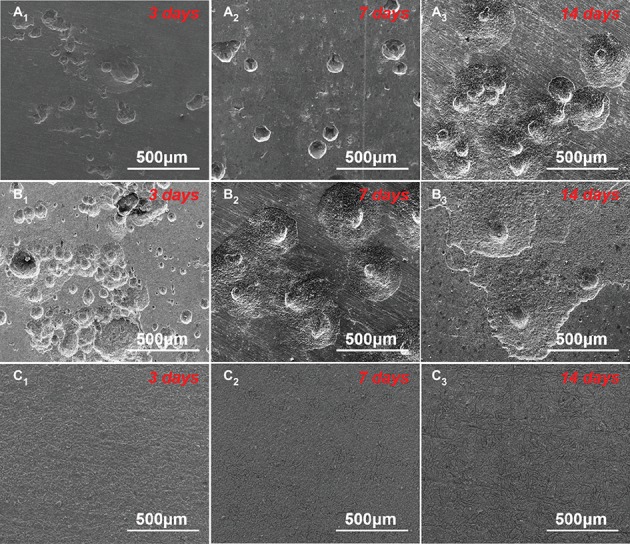
SEM images of sample surfaces after removing the corrosion products after 3 **(A_1_–C_1_)**, 7 **(A_2_–C_2_)**, and 14 days **(A_3_–C_3_)** of immersion in **(A)** 0.5 ppm, **(B)** 3.0 ppm, and **(C)** 5.0 ppm inoculated media.

**Figure 5:**
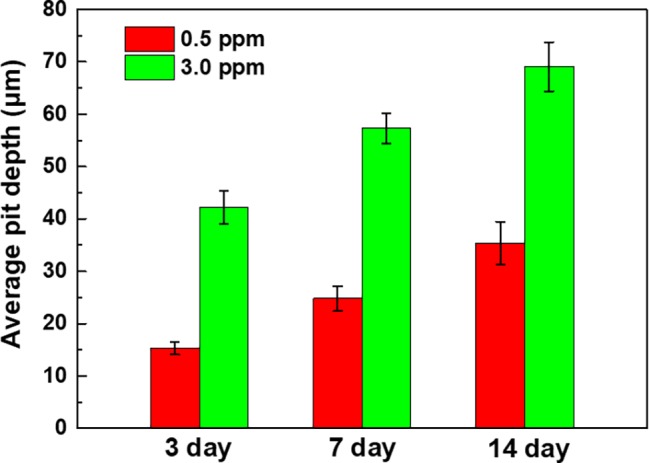
Average pit depths of the samples after 3, 7, and 14 days of immersion in 0.5 ppm and 3.0 ppm inoculated media.

**Figure 6:**
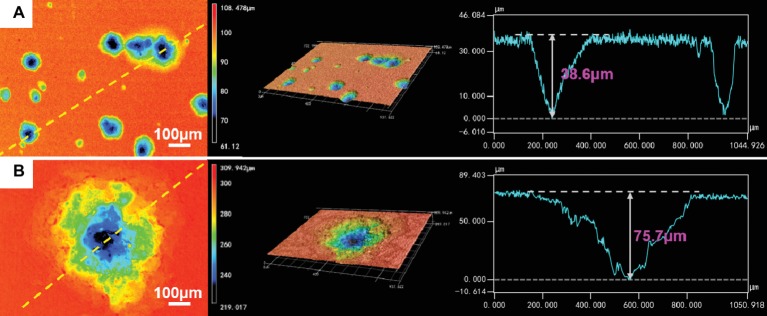
CLSM images and maximum pit depths of the samples after 14 days of immersion in **(A)** 0.5 ppm and **(B)** 3.0 ppm inoculated media.

The corrosion morphologies of the samples after removing the corrosion products are shown in Figure 4. For the samples in 0.5 ppm inoculated media, the sample surfaces exhibited obvious localized corrosion behavior. With the increase of time, the localized corrosion was gradually aggravated, and the pit depths were gradually increased. After 14 days of immersion, the average pit depth increased to ~35 μm (Figure 5), and the maximum pit depth reached 38.6 μm (Figure 6). In 3.0 ppm inoculated media, the localized corrosion was more serious. After 14 days, the average depth of localized corrosion pits reached ~70 μm, and the maximum depth was 75.7 μm (Figure 6), which was almost twice that of the maximum depth in 0.5 ppm inoculated media. Compared with the samples in 0.5 ppm inoculated media, the number of corrosion pits decreased in 3.0 ppm inoculated media, which was mainly caused by the combination of adjacent corrosion pits during their expansion process. When the DOC was further elevated to 5.0 ppm, it is worth noting that the sample surfaces showed apparent uniform corrosion characteristics, and no localized corrosion pit could be observed. It suggests that with the increase of DOC from 0.5 to 3.0 ppm, the localized corrosion of carbon steel was promoted. Subsequently, it was replaced by uniform corrosion when the DOC was as high as 5.0 ppm. Compared with the samples in sterile media, the samples in inoculated media exhibited totally different corrosion morphologies.

### Weight Loss Analysis

**Figure 7:**
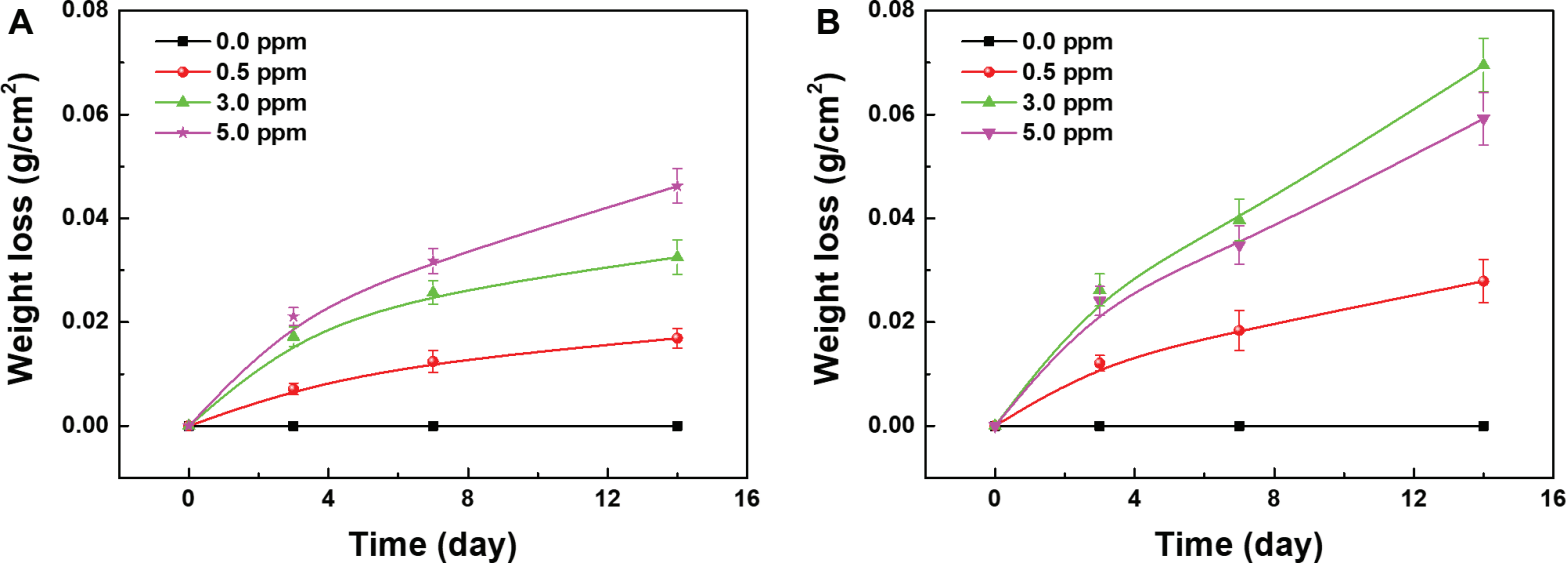
Weight loss of the samples after 3, 7, and 14 days of immersion in **(A)** sterile media and **(B)** inoculated media with different DOCs.

The weight loss measurements of carbon steel samples were also carried out to quantitatively analyze the influence of DOC on the MIC of carbon steel. Figure 7A shows the weight loss results of the samples in different sterile media after 3, 7, and 14 days of immersion. With the increase of DOC, the corrosion weight loss of carbon steel samples increased gradually, and the weight loss of carbon steels in anaerobic media remained at zero during 14 days. Under sterile condition, the increase of DOC promoted the uniform corrosion of carbon steel samples.

The weight loss results of the samples in different inoculated media after 3, 7, and 14 days of immersion are shown in Figure 7B. During 14 days, the corrosion weight loss of all samples increased gradually with time. With the increase of DOC, the weight loss of carbon steels increased first and decreased thereafter, reaching the maximum value in 3.0 ppm inoculated medium. After 14 days, the corrosion weight loss of carbon steel in 3.0 ppm inoculated medium was approximately 0.07 g, which was about 1.5 times as much as the maximum weight loss in sterile media (Figure 7A). These results indicates that with the increase of DOC, the MIC of carbon steels was first aggravated and then relieved, and the MIC was the most serious in 3.0 ppm inoculated medium.

### Electrochemical Analysis

**Figure 8:**
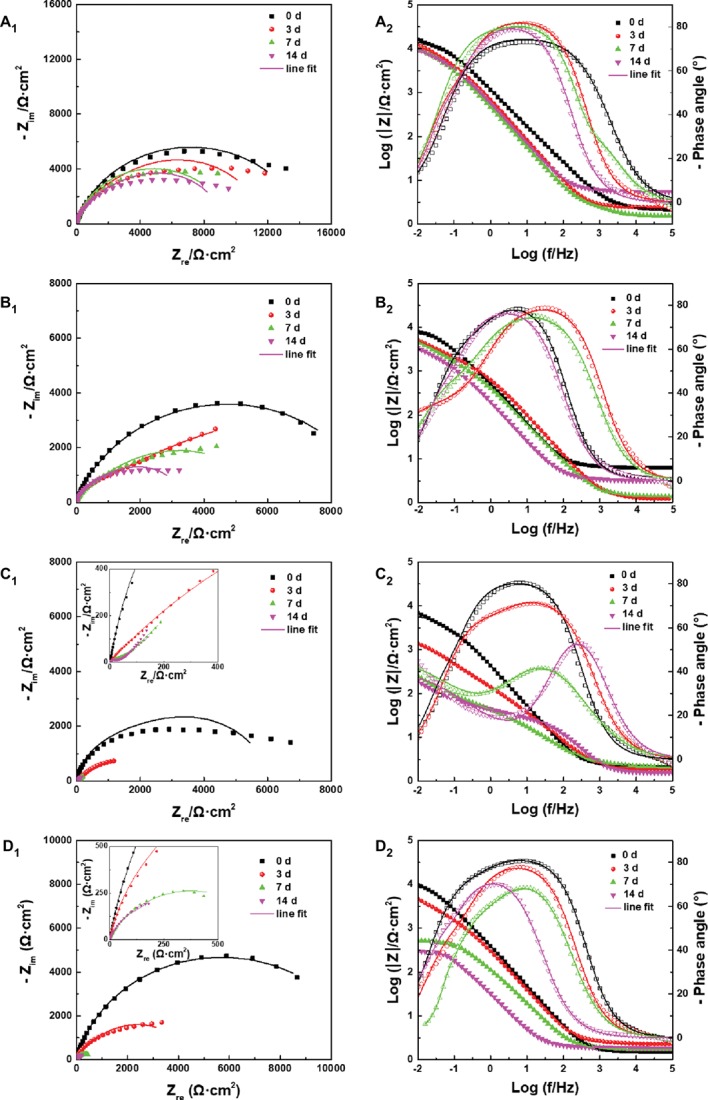
**(A_1_–D_1_)** Nyquist plots and **(A_2_–D_2_)** Bode plots of the samples after different days of immersion in inoculated media with the DOC of **(A)** 0.0 ppm, **(B)** 0.5 ppm, **(C)** 3.0 ppm, and **(D)** 5.0 ppm.

The effect of archaeon *N. tibetense* on the corrosion electrochemical process of carbon steel was examined by EIS and potentiodynamic polarization measurements. The evolution of EIS results of the samples exposed in different inoculated media with time was compared and presented in Figure 8. For different inoculated media, the radii of capacitive arc in low frequency region decreased gradually with time. In corresponding Bode plots, the impedance modulus in low frequency region also decreased gradually. With the increase of DOC, the decline rate of impedance increased first and then decreased, which reached the fastest in 3.0 ppm inoculated medium.

**Figure 9:**
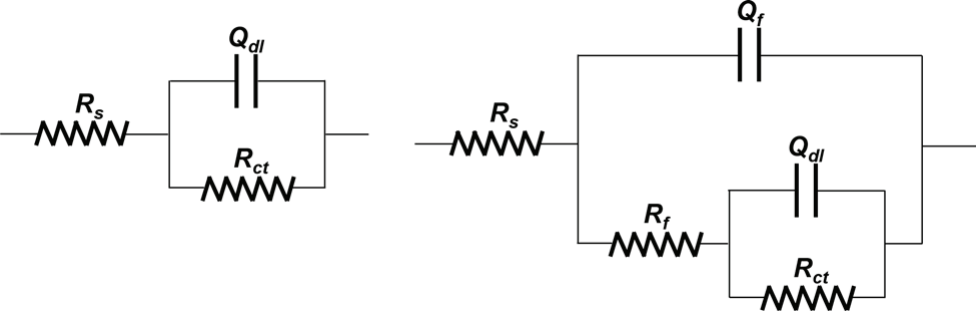
Electrical equivalent circuit used for fitting EIS results.

The EIS plots were further fitted by the electrical equivalent circuits in Figure 9. The electrical equivalent circuit with one time constant (Figure 9A) was used to fit the EIS results in Figure 8A. The other EIS results were fitted using the electrical equivalent circuit in Figure 9B. *Q_f_* and *Q_dl_* represented the constant phase element (CPE) of the mixed film of corrosion products and biofilms and the electric double layer, respectively. *R_f_* and *R_ct_* were the resistance of the mixed film and the charge transfer resistance, respectively. The CPE was used to replace the ideal electrical capacitance for more precise fitting (Zhang et al., 2016; Qian et al., 2017b). The *R_ct_* values after 3, 7, and 14 days of immersion in different inoculated media were listed in Table 1, which were used to represent the variation of corrosion resistance during the immersion. The variation of the *R_ct_* values exhibited a similar trend to the impedance modulus in low frequency region. After 14 days, the samples in 3.0 ppm inoculated medium had the minimum *R_ct_* values. It also indicates that the samples in 3.0 ppm inoculated medium had the fastest electron transfer rate at the metal/medium interface and the maximum corrosion rate.

**Table 1:** The *R_ct_* values of the samples in inoculated media with different DOCs (kΩ cm^2^).

	0.0 ppm	0.5 ppm	3.0 ppm	5.0 ppm
0 day	9.23 ± 0.36	8.52 ± 0.48	6.75 ± 0.37	8.64 ± 0.42
3 days	9.06 ± 0.29	4.04 ± 0.27	1.23 ± 0.19	3.31 ± 0.24
7 days	8.98 ± 0.17	3.69 ± 0.15	0.16 ± 0.03	0.22 ± 0.03
14 days	8.81 ± 0.23	3.14 ± 0.18	0.12 ± 0.04	0.19 ± 0.02

**Figure 10:**
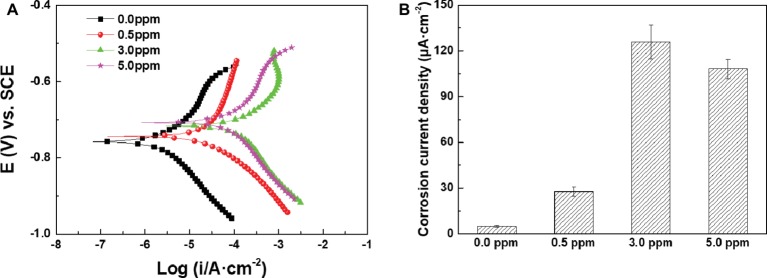
**(A)** Potentiodynamic polarization curves and **(B)** I*_corr_* values of the samples after 14 days of immersion in inoculated media with different DOCs.

Figure 10A shows the potentiodynamic polarization curves of the samples after 14 days of immersion in inoculated media with different DOCs, and corresponding *I_corr_* values were plotted in Figure 10B. *I_corr_* values first increased and then decreased with the increase of DOC, which showed the same variation trend with the results of weight loss tests. The electrochemical results further verify that the effect of DOC on the MIC of carbon steel can be divided into two stages. Before 3.0 ppm, the increase of DOC decreased the *R_ct_* and increased the *I_corr_*, which promoted the MIC of carbon steel. On the contrary, from 3.0 to 5.0 ppm, the increase of DOC increased the *R_ct_* and reduced the *I_corr_*, thus relieving the MIC to a certain extent.

### Corrosion Product Analysis

**Figure 11:**
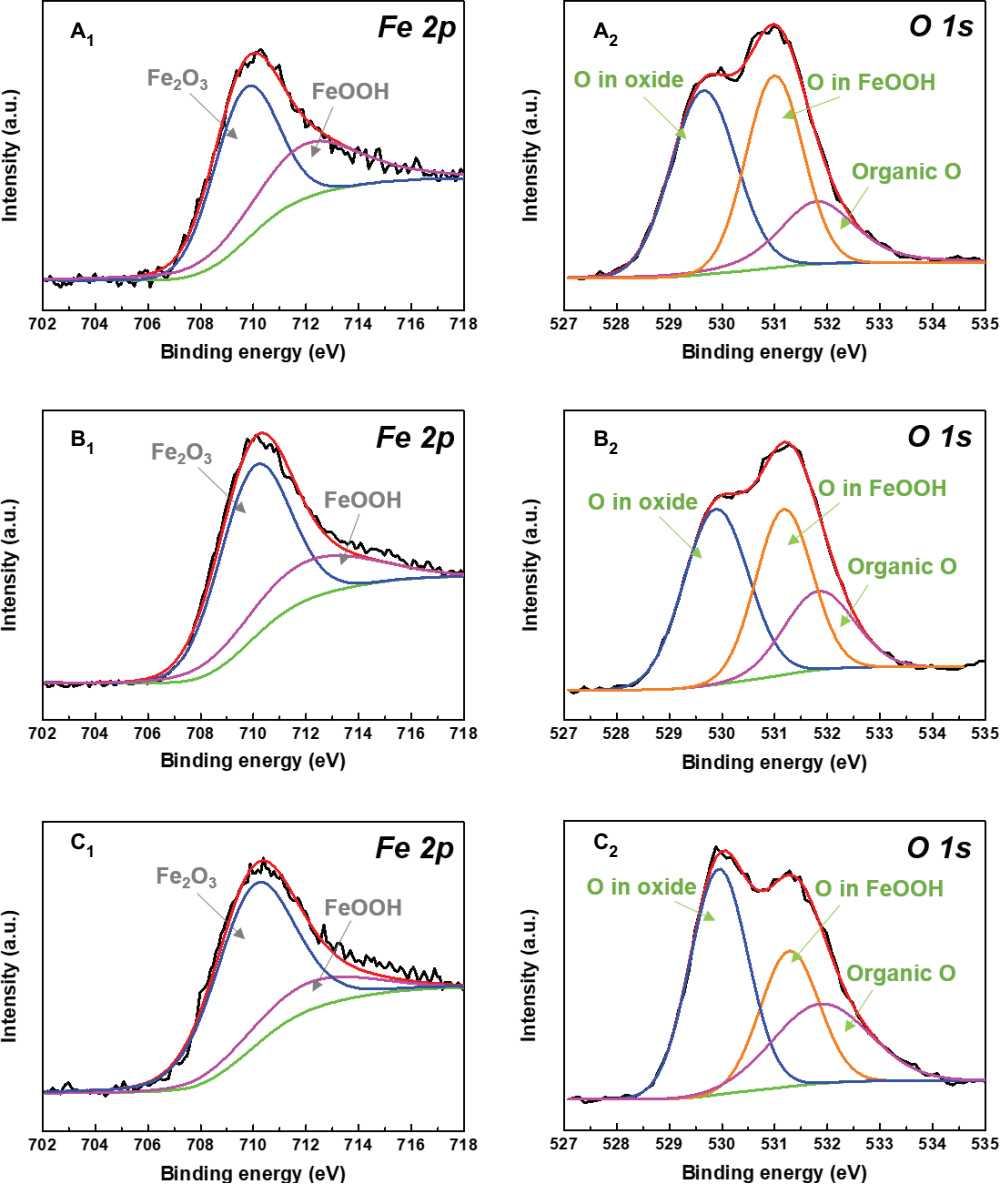
XPS spectra of Fe 2p **(A_1_–C_1_)** and O 1s **(A_2_–C_2_)** of the corrosion products after 14 days of immersion in **(A)** 0.5 ppm, **(B)** 3.0 ppm, and **(C)** 5.0 ppm inoculated media.

XPS was used to further analyze the compositions of corrosion products on sample surfaces after 14 days of immersion in different inoculated media. High-resolution XPS spectra for Fe and O elements of different corrosion products are shown in Figure 11. The Fe 2p spectra for different corrosion products were composed of two peaks at 710.0 and 712.0 eV, which were assigned to the Fe_2_O_3_ and FeOOH, respectively (Tourabi et al., 2013; Zarrouk et al., 2015). Fe_2_O_3_ and FeOOH were generated by the chemical reactions below (Morcillo et al., 2014):

(1) $$4{Fe}^{2+}+4H_{2}O+O_{2}\to 2{Fe}_{2}O_{3}+8H^{+}$$

(2) $$4{Fe}^{2+}+6H_{2}O+O_{2}\to 4\mathrm{FeOOH}+8H^{+}$$

It indicates that the change of DOC in inoculated media did not affect the compositions of corrosion products. With the increase of DOC, the relative amount of loose Fe_2_O_3_ increased gradually, while the relative amount of FeOOH decreased. All O 1s spectra were fitted with three peaks at 529.9, 531.2, and 531.8 eV, respectively. The fitted peaks at 529.9 eV were attributed to the O in oxides, which enhanced with the increase of DOC. This result implied that the proportion of Fe_2_O_3_ in the mixed films on sample surfaces increased. The peaks located at 531.8 eV were related to the organic O in biofilms (Liu et al., 2017). The relative amount of organic O also increased with the DOC, suggesting that the proportion of biofilm in the mixed films on sample surfaces also increased.

## Discussion

According to previous research (Qian et al., 2018), it is clear that the dissolved oxygen plays two roles in the MIC of carbon steel by halophilic archaeon *N. tibetense*. First, dissolved oxygen acts as the electron acceptor for cell respiration and growth, which determines the maximum number of *N. tibetense* cells in the culture medium (Figure 1). The archaeon *N. tibetense* has been shown to be able to consume metallic iron as an energy source (Qian et al., 2018), so the number of *N. tibetense* cells in the culture medium will directly affect the dissolution rate of metallic iron. At the same time, dissolved oxygen also functions as the cathode depolarizer that affects the electrochemical corrosion process on metal/medium interface, and its uneven distribution easily leads to the generation of oxygen concentration cell, which is considered to be the main reason for the formation of localized corrosion (Little and Lee, 2014). The coupling of these two roles controls and accelerates the corrosion of carbon steel in *N. tibetense*-inoculated culture medium. The change of DOC will inevitably affect these two aspects and accordingly affect the MIC behavior of carbon steel.

Carbon steels in the sterile media with different DOCs mainly suffered from uniform corrosion (Figures 2A_2_–C_2_). By contrast, the typical localized corrosion behaviors appeared in the inoculated culture media (Figure 4), which were undoubtedly caused by the introduction of archaeon *N. tibetense*. Microbial cells are prone to inhomogeneous attachment on metal surface, which is more conducive to energy sharing and resistance to harsh living environment (Zhang et al., 2015), and this behavior is also related to the type and microstructure of metal materials (Javed et al., 2013, 2015). This inhomogeneous attachment can easily lead to the formation of discontinuous biofilms on metal surface. As an important component of biofilm, extracellular polymeric substance (EPS) is mainly composed of macromolecules such as polysaccharides, lipids, and proteins (Beech and Sunner, 2004), which is harmful to the diffusion of dissolved oxygen to metal surface (Moradi et al., 2015; Liu et al., 2015a). Hence, oxygen-poor areas will be formed below the biofilms, while oxygen supply is relatively sufficient in areas without biofilm, thus forming oxygen concentration cell. As shown in Figures 3, 4, the carbon steel surfaces under the biofilms became anode areas and occurred in the dissolution of metallic iron, which lead to the localized corrosion and generated corrosion pits. Besides, the direct consumption of carbon steel by archaeon *N. tibetense* can also strengthen the concentration cell to a certain extent.

According to the growth curves and SEM observations, it can be determined that the change of DOC in the culture media had a significant effect on the concentrations of *N. tibetense* cells in the culture media, which further affected the morphologies and distributions of biofilms on carbon steel surfaces at the initial stage of immersion. In case of insufficient oxygen supply (0.5 ppm), the number of suspended cells in 0.5 ppm medium was very small, which resulted in a small amount of cells, and small scale of biofilms adhered on the metal surface at the initial immersion stage (Figure 3A_1_). Although the oxygen concentration cell could promote the localized corrosion, the insufficient oxygen supply in the cathode areas became the limiting factor of corrosion. In addition, the limited number of *N. tibetense* cells in the culture medium could not cause rapid dissolution of metallic iron. Hence, the localized corrosion of carbon steel in 0.5 ppm medium was relatively weak, and the scale of corrosion product clusters and the size of corrosion pits were small.

Once the DOC increased to 3.0 ppm, the cathodic oxygen reduction of concentration cell was accelerated, which promoted the effect of oxygen concentration cell. At the same time, the significantly increased number of *N. tibetense* cells required more energy supply, which also accelerated the consumption and dissolution of metallic irons in the anode areas of oxygen concentration cells. Therefore, compared with the samples in 0.5 ppm medium, the localized corrosion of the samples in 3.0 ppm medium was evidently aggravated, and the size of corrosion product clusters and corrosion pits increased rapidly during 14 days of immersion. In the 0.5 and 3.0 ppm media, the corrosion of carbon steels mainly depended on the coupling effect of oxygen concentration cell and direct consumption of metallic iron by *N. tibetense* cells.

However, if the DOC was as high as 5.0 ppm, the corrosion behavior of carbon steel was significantly different (Figure 3C). The limited organic matters in the culture medium could not meet the needs of the rapid proliferation of *N. tibetense* cells, which forced a large number of cells to adhere to the surface of carbon steel to obtain energy for survival. Abundant *N. tibetense* cells made most of the metal surface covered by biofilms, which weakened the inhomogeneous distribution of dissolved oxygen on the metal surface. Hence, it was hard to form the oxygen concentration cell, and the sample surface mainly suffered from uniform corrosion, as shown in Figure 4C. The increased proportion of biofilm in the mixed film on the carbon steel surface enhanced the inhibition effect of oxygen diffusion to the carbon steel surface. Therefore, compared with the samples in 3.0 ppm medium, the corrosion of carbon steel in 5.0 ppm medium was relatively weak. In 5.0 ppm medium, the corrosion of carbon steel mainly depended on the consumption of metallic iron by archaeon *N. tibetense*.

## Conclusion

In this work, we studied the effect of DOC on MIC behavior of Q235 carbon steel caused by halophilic archaeon *N. tibetense*. The carbon steel samples were immersed into the culture media with different DOCs for immersion tests. Through corrosion morphology observation, weight loss tests, electrochemical tests, and corrosion product analysis, the following conclusions were obtained in this study:

1. As the DOC increased from 0.0 to 5.0 ppm, the weight loss of carbon steel first increased and then decreased, and localized corrosion behavior was gradually aggravated and then replaced by uniform corrosion behavior. The samples in 3.0 ppm inoculated medium exhibited the most serious MIC.
2. When the DOC increased from 0.0 ppm to 3.0 ppm, the increase of DOC strengthened the oxygen concentration cell by promoting cathodic reaction, and the increase of cell number in the culture media consumed more metallic iron as energy source. The coupling of these two aspects promoted the localized corrosion with the increase of DOC.
3. When the DOC reached 5.0 ppm, the uniformly distributed biofilms on the metal surface led to uniform corrosion. Moreover, the increased proportion of biofilm on carbon steel surface enhanced the inhibition effect of oxygen diffusion. Therefore, the MIC of carbon steel is weaker than that in 3.0 ppm inoculated medium.

## Author Contributions

HQ designed and performed the experiments. YH, ZL, LH, and YL assisted in performing some experiments. HQ wrote the manuscript. DZ, LM, and CD revised the manuscript. All authors read and approved the submitted manuscript.

### Conflict of Interest Statement

The authors declare that the research was conducted in the absence of any commercial or financial relationships that could be construed as a potential conflict of interest.
